# The roles of lncRNA in hepatic fibrosis

**DOI:** 10.1186/s13578-018-0259-6

**Published:** 2018-12-06

**Authors:** Hu Peng, Lin-Yan Wan, Jia-Jie Liang, Yan-Qiong Zhang, Wen-Bing Ai, Jiang-Feng Wu

**Affiliations:** 1 Medical College, China Three Gorges University, 8 Daxue Road, Xiling District, Yichang, 443002 China; 2 Digestive Medicine, The People’s Hospital of China Three Gorges University, 31 Huti Subdistrict, Xi Ling District, Yichang, 443000 Hubei China; 3 Institute of Organ Fibrosis and Targeted Drug Delivery, China Three Gorges University, 8 Daxue Road, Xiling District, Yichang, 443002 China; 4 Hubei Key Laboratory of Tumor Microenvironment and Immunotherapy, China Three Gorges University, 8 Daxue Road, Xiling District, Yichang, 443002 China; 5 The Yiling Hospital of Yichang, 31 Donghu Road, Yi Ling District, Yichang, 443100 Hubei China

**Keywords:** Long non-coding RNAs, Hepatic fibrosis, Hepatic stellate cell, TGF-β signaling pathway, DNA methylation, ceRNA

## Abstract

Increasing evidence indicates that long non-coding RNAs (lncRNAs) regulate gene or protein expression; however, their function in the progression of hepatic fibrosis remains unclear. Hepatic fibrosis is a continuous wound-healing process caused by numerous chronic hepatic diseases, and the activation of hepatic stellate cells (HSCs) is generally considered to be a pivotal step in hepatic fibrosis. In the process of hepatic fibrosis, some lncRNAs regulates diverse cellular processes. Here are several examples: the lncRNA metastasis-associated lung adenocarcinoma transcript 1 (MALAT1) and liver fibrosis-associated lncRNA1 (lnc-LFAR1) promote HSC activation in the progression of hepatic fibrosis via the transforming growth factor-β signaling pathway; the lncRNA HIF 1 alpha-antisense RNA 1 (HIF1A-AS1) and Maternally expressed gene 3 reduce HSC activation which are associated with DNA methylation; the lncRNA plasmacytoma variant translocation 1, Homeobox (HOX) transcript antisense RNA and MALAT1 promote HSC activation as competing endogenous RNAs (ceRNAs); the long intergenic non-coding RNA-p21 (lncRNA-p21) and Growth arrest-specific transcript 5 reduce HSC activation as ceRNAs. As we get to know more about the function of lncRNAs in hepatic fibrosis, more and more ideas for the molecular targeted therapy in hepatic fibrosis will be put forward.

## Background

Hepatic fibrosis is a continuous wound-healing process that results in the dysregulation of extracellular matrix (ECM) proteins and the distortion of normal liver architecture [1]. Many chronic hepatic diseases, such as viral hepatitis, alcohol toxicity, drug abuse, metabolic syndrome, hereditary disorders of metabolism, autoimmune hepatitis and *Clonorchis sinensis* infection, lead to hepatic fibrosis and even cirrhosis [2], which is the primary stage of hepatic carcinoma, leading to one of the major causes of mortality in cancer worldwide.

Although extensive studies on hepatic fibrosis have been reported, their regulatory mechanisms are still partially understood. The activation of hepatic stellate cells (HSCs), the resident perisinusoidal cell type, is generally considered to be a pivotal step in hepatic fibrosis [3]. In normal hepatic tissue, HSCs with abundant vitamin A stores are quiescent. Following with hepatic injury of any etiology, the quiescent HSCs lose their stored vitamin A and trans-differentiate into fibrogenic myofibroblast-like cells. The activated HSCs are identified as proliferative cells that express ECM, and secrete profibrogenic mediators, thereby contributing to the fibrosis [4]. Therefore, the suppression of the HSC activation is regarded to be a potential therapeutic target for hepatic fibrosis.

Genome tiling arrays and cap analysis gene expression showed that non-protein coding RNAs (ncRNAs), which were considered to be ‘‘evolutionary junk” in the past, have more functions in transcription [5, 6]. Recently, a large number of ncRNA molecules have been identified by RNA microarrays and next-generation sequencing of transcriptomes [7]. NcRNAs are classified into two types based on their relative sizes. Those less than 200 nucleotides (nt) are called small or short non-coding RNAs, while those longer than 200 nt are called long non-coding RNAs (lncRNAs) [8]. LncRNAs are considered to play roles in physiological conditions as well as in several human diseases, including cancer, metabolic diseases, cardiovascular diseases and so on [9]. Increasing evidence has suggested that lncRNAs regulate gene or protein expression by coordinating epigenetic, transcriptional, or post-transcriptional processes [10]. But the function of lncRNAs in hepatic fibrosis remains elusive.

**Fig. 1:**
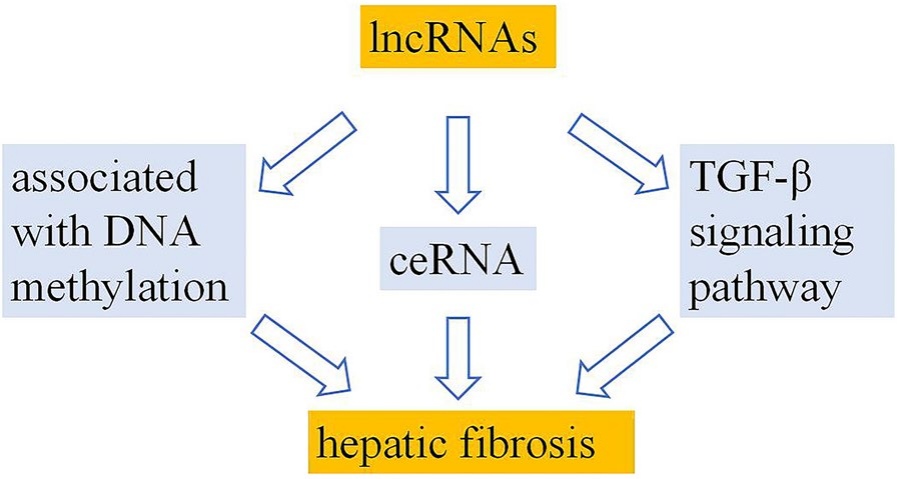
It is the summary of the mechanism of lncRNAs that regulate hepatic fibrosis through TGF-β signaling pathway, DNA methylation and ceRNA in this review

The goal of this review is to summarize the roles of lncRNAs in hepatic fibrosis, including the regulation on transforming growth factor (TGF)-β signaling pathway, DNA methylation and competing endogenous RNAs (ceRNAs) (Fig. 1) according to current knowledge. It will illustrate some important information for the treatment hepatic fibrosis and present novel guidance in future researches.

### The interaction of lncRNAs and the transforming growth factor (TGF)-β signaling pathway in hepatic fibrosis

TGF-β is a key regulator of liver physiology and pathology during the process of initial liver injury-inflammation-fibrosis [11]. TGF-β1, a potent fibrogenic cytokine from the autocrine or paracrine pathway, is a crucial signal that promotes HSC activation [12]. Some studies have shown that lncRNAs interact with the TGF-β signaling pathway to promote HSC activation and then induce hepatic fibrosis.

**Fig. 2:**
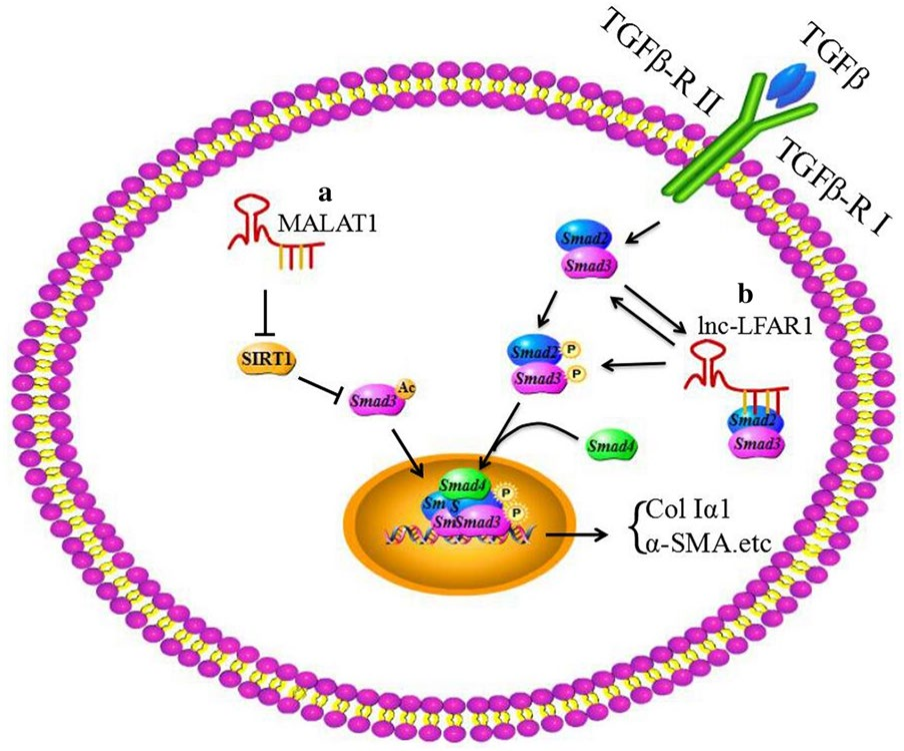
lncRNAs regulate hepatic fibrosis via TGF-β signaling pathway. **a** MALAT1 represses SIRT1 to inhibit the deacetylation of Smad3. Then the deacetylation of Smad3 binds to fibrogenic genes, such as colIα1, to induce the expression of colIα1. **b** lnc-LFAR1 is induced by Smad2/3 and in turn to promote the phosphorylation of Smad2/3, that provides a positive feedback loop to enhance Smad2/3 binding to the target gene, therefore causing the high expression of colIα1 and α-SMA. The deposition of ECM and activation of HSCs contribute to the hepatic fibrosis

Metastasis-associated lung adenocarcinoma transcript 1 (MALAT1) which is located in human chromosome 11q13.1 (mouse chromosome 19qA), also known as nuclear-enriched abundant transcript 2 (NEAT2), is a widely expressed lncRNA, and was firstly identified through subtractive hybridization in stage I of non-small cell lung cancer [13, 14]. A growing number of evidence indicated that MALAT1 was closely related to various pathological processes, including diabetes complications and hepatic carcinoma [15], and could influence the progression of hepatic fibrosis by repressing the expression and function of silent information regulator 1 [SIRT1, a Nicotinamide adenine dinucleotide (NAD)-dependent class III protein deacetylase] [16]. As one of the best characterized deacetylase enzymes, SIRT1 can protect cultured cells against metabolic, geneotoxic, hypoxic, and heat stress by deacetylating a number of key transcription factors [17], while it can induce the deacetylation of Smad3 (a downstream mediator of TGF-β signaling pathway) and weaken the ability of Smad3 binding to the promoter of fibrogenic genes, such as collagen type I gene promoters, which means that the activation of SIRT1 attenuates TGF-β signaling and then reduces TGF-β-stimulated collagen expression [18, 19]. In summary, MALAT1 can promote the HSC activation through blocking the SIRT1 mediated inhibition of TGF-β signaling pathway in the progression of hepatic fibrosis (Fig. 2a). What’s more, MALAT1 is also reported that it acts as a competing endogenous RNA for miR-101b to regulate RAS-related C3 botulinum substrate 1 (Rac1) and contributes to hepatic fibrosis [20] (Fig. 4).

The liver fibrosis-associated lncRNA1 (lnc-LFAR1) is a 734 nt transcript and it was originally identified as a liver-enriched lncRNA in fibrotic liver of mice. The lnc-LFAR1 of mice is located in chromosome 4q25, and it adjacents to the CYP2U1 and HADH genes which are the same as human [21]. ALGGEN-PROMO and JASPAR software analysis have shown that there are three potential Smad2/3 binding sites (SBE) in the promoter of lnc-LFAR1, which means that Smad2/3 can bind to the promoter of lnc-LFAR1 to increase its expression. Furthermore, the lnc-LFAR1 in turn up regulates the expression of Smad2/3 and promotes Smad2/3 phosphorylation in liver fibrogenesis [21]. The phosphorylation of Smad2/3 promotes its nuclear translocation and the ability binding to the target promoters, such as collagen type I gene [22]. All in all, lnc-LFAR1 induces the activation of HSCs to promote hepatic fibrosis by interacting with TGF-β signaling pathway (Fig. 2b).

### lncRNA associates with DNA methylation to inhibit the activation of HSCs in hepatic fibrosis

DNA methylation is a type of epigenetic modification in mammals and involves in numerous biological processes, including transposable element silencing, genomic imprinting and X chromosome inactivation [23]. The process of DNA methylation is regulated by methyltransferases, for examples, DNA methyltransferases (DNMT1, DNMT3a, DNMT3b) induce de novo methylation and ten–eleven translocation methylcytosine dioxygenase (TET) family member enzymes (TET1, 2 and 3) induce DNA demethylation re-activated or re-expressed silenced genes. DNMTs and TETs are critical in the cycle of DNA methylation and demethylation [24]. Advancing studies indicate that DNMTs and TETs play significant roles to change 5-methylcytosine and 5-hydroxymethylcytosine during HSC transdifferentiation to myofibroblast-like cells [25]. Increasing evidence shows that several lncRNAs are associated with DNA methylation to inhibit the activation of HSCs in hepatic fibrosis.

**Fig. 3:**
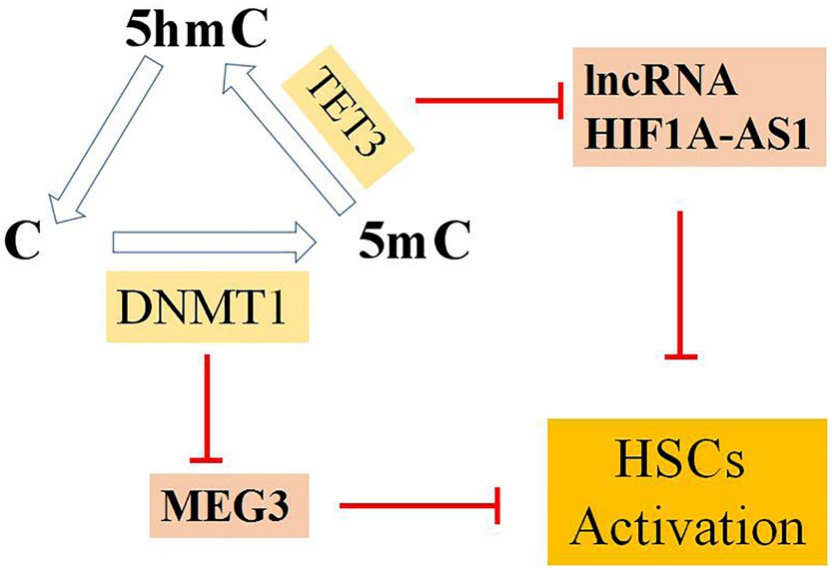
The transform among Cytosine (C), 5-methylcytosine (5-mC) and 5-hydroxymethylcytosine (5-hmC) forms the basis of the cycle of DNA methylation and demethylation. As the critical regulation enzymes in the cycle of DNA methylation and demethylation, DNMT1 and TET3 contribute to hepatic fibrosis through repressing MEG3 and lncRNA HIF1A-AS1

Maternally expressed gene 3 [MEG3, which is also known as gene trap locus 2 (GTL2)], is a lncRNA with the length of 1.6 kb nucleotides. It is a part of the DLK1–MEG3 imprinting locus and located at human chromosome 14q32 and at mouse distal chromosome 12 [26]. It is expressed in virous human tissues and acts as a tumor suppressor [27]. Recently, the loss of MEG3 expression has been gradually proved in various types of human cancers, such as hepatic cancer, gastric cancer, lung cancer, glioma, cervical cancer, bladder cancer [28–34]. On the one hand, MEG3 can selectively regulate p53 target gene expression resulting in the accumulation of p53 protein, and leading to cell growth inhibition [35]; on the other hand, MEG3 activates p53, and then intervenes in the p53-dependent mitochondrial apoptosis pathway to increase mitochondrial cytochrome c release and to culminate in direct caspase activation [35]. The expression of MEG3 was negatively correlated with the differentially methylated regions (DMRs) hypermethylation level, suggesting that DNA methylation plays an important role in silencing the MGE3 gene [28]. DNA methyltransferase 1 (DNMT1) can maintain methylation pattern on the daughter strand after DNA methylation and contribute to hypermethylation of MEG3 gene promoter and decrease the expression of MEG3 [30]. MEG3 could activate p53 to cause caspase-3-dependent apoptosis and reduce the expression of alpha-1 type I collagen (colIα1) and α-smooth muscle actin (α-SMA) in activeted HSCs induced by TGF-β1 [36]. It is indicated that MEG3 plays a critical role in HSC activation and hepatic fibrogenesis (Fig. 3). Therefore, it reveals that high-expression of MEG3 are potentially regarded as a novel therapeutic target for treating liver fibrosis.

The lncRNA HIF 1 alpha-antisense RNA 1 (HIF1A-AS1) was initially reported in human kidney cancers and it is located on chromosome 14, with 2100 nt [37]. It has been demonstrated that the HIF1A-AS1 involves in the proliferation and apoptosis of vascular smooth muscle cells and the vascular endothelial cells [38, 39], and it is also related with the process of the non-small cell lung cancer and the colorectal carcinoma [40, 41]. HIF1A-AS1 acts as an inhibitor or an activator of cell proliferation and apoptosis depending on its binding partners and cell types. As one of the ten to eleven translocation (TET) family members, TET3 can catalyze 5-methylcytosine (5-mC) demethylate to 5-hydroxymethylcytosines (5-hmCs), which leads to cancer suppression [42]. In hepatic fibrosis, TET3 promotes the activation of HSCs through suppressing the expression of 1ncRNA1A-AS1 [43]. Thus, lncRNA HIF1A-AS1 interacts with the partner TET3 associated with DNA methylation to inhibit the activation of HSCs. These indicate that the up-regulation of lncRNA HIF1A-AS1 may be a potential therapy pathway for hepatic fibrosis (Fig. 3).

### lncRNAs act as ceRNAs in hepatic fibrosis

MicroRNAs (miRNAs) pair with miRNA response elements (MREs) on target RNA transcripts resulting in degradation or translational repression of the target transcripts [44]. A competing endogenous RNA (ceRNA) is a endogenous origin transcript targeted by a miRNA that sequesters the activity of the bound miRNA, effectively de-repressing other targets of that miRNA [45]. LncRNAs have been gained substantial attention as ceRNAs to sponge miRNAs to consequently modulate the derepression of miRNA targets, thereby protecting their target mRNAs [46]. Recently, several studies have shown that lncRNAs act as ceRNAs which play an important regulatory role in the process of hepatic fibrosis.

The long intergenic non-coding RNA-p21 (lncRNA-p21), which resides 15 Kb upstream of the gene encoding the critical cell cycle regulator Cdkn1a (also known as p21), contains two exons comprising 3.1 Kb [47]. LncRNA-p21 functions as a downstream transcriptional repressor in the p53 pathway via activating p53 to promote apoptosis [48]. It has been reported that lncRNA-p21 has been deregulated in various human diseases, such as skin tumors, prostate cancer and hepatocellular carcinoma [49–51]. It also acts as a tumor suppressor in cancers, but the mechanism of the process remains unclear. LncRNA-p21 directly binds target mRNA to regulate the translation as a post-transcriptional inhibitor [52], while it also acts as a locus-restricted coactivator for p53-mediated p21 expression in regulating the G1/S checkpoint [53]. Furthermore, it has been proposed that the lncRNA-p21 is also able to regulate gene expression by directing the chromatin localization of protein binding partners [54]. As a tumor suppressor, the phosphatase and tensin homologue deleted on chromosome 10 (PTEN) is often deregulated in various cancers, and it is a direct target of miR-181b that has been reported in hepatic fibrosis [55]. Yu et al. demonstrated that the lncRNA-p21 enhanced PTEN expression through competitively binding miR-181b as a ceRNA and inhibited the activation of HSCs via PTEN/Akt pathway in hepatic fibrosis [56]. It is also reported that the lncRNA-p21 sponges miR-17-5p to inhibit WIF1 through Wnt/β-catenin pathway resulting in suppression the HSC activation [57]. According to the research results above, we conclude that the lncRNA-p21 acts as a ceRNA to prevent the HSC activation in hepatic fibrosis.

Growth arrest-specific transcript 5 (GAS5) was initially discovered in a screen for potential tumor suppressor genes which expressed at high levels during growth arrest and it was originally isolated from mouse embryo NIH 3T3 cells using subtraction hybridization [58]. GAS5 has been reported as a tumor suppressor in some kinds of cancers, and it has been shown the GAS5 is involved in proliferation, apoptosis, and migration of tumor cells in breast cancer, gastric cancer and prostate cancer [59–61]. The GAS5 directly binds miR-21 to down-regulate its expression at exon 4 of GAS5 and negatively regulate the expression of miR-21 in hepatocellular carcinoma [62]. Moreover, it was also demonstrated that the GAS5 acts as ceRNA to control cardiac fibroblast activation and cardiac fibrosis by targeting miR-21 through PTEN/MMP-2 signaling pathway [63]. In hepatic fibrosis, the GAS5 through interacting with miR-222 to promote the expression of p27 protein, thereby inhibiting the activation and proliferation of HSCs [64].

Plasmacytoma variant translocation 1 (PVT1) in size of > 300 nt is transcribed from a locus adjacent to the MYC locus on human chromosome 8q24 (mouse chromosome 15) [65]. Recently, the PVT1 is found to be up-regulated in a series of human tumors, such as hepatocellular carcinoma, ovarian cancer, malignant pleural mesothelioma, non-small lung cancer and renal cancer [66–70]. PVT1 was deemed as a mediator of ECM in the diabetic kidney [71], which suggested that the PVT1 might involve in fibrosis. Epithelial–mesenchymal transition (EMT) process is considered as a key event in the activation of HSCs and hepatic fibrosis via activating Hedgehog (Hh) signaling pathway [72]. Patched1 (PTCH1), a member of Hh family, is also a negative regulator of Hh pathway. PVT1 can indirectly enhance PTCH1 methylation and down-regulate the expression of PTCH1 via competitively binding miR-152. Therefore, the PVT1 may serve as a ceRNA for miR-152 through Hh pathway to regulate the activation of HSC in hepatic fibrosis [65].

Homeobox (HOX) transcript antisense RNA (HOTAIR) is a 2158 nt lncRNA that locates to a boundary of the HOXC locus, one of the four chromosomal loci (HOX A to D) containing the clustered HOX genes [73]. Accumulating studies have indicated that HOTAIR is up-regulated in multiple cancers, including breast cancer, lung adenocarcinoma, renal cell carcinoma, pancreatic cancer, hepatocellular carcinoma [74–78]. MiR-29b can up-regulate the expression of PTEN via DNMT3b to suppress liver fibrosis [79]. The HOTAIR acts as a ceRNA to sponge miR-29b and then attenuates DNMT3b, leading to enhancement of PTEN methylation that contributes to liver fibrosis [80].

**Fig. 4:**
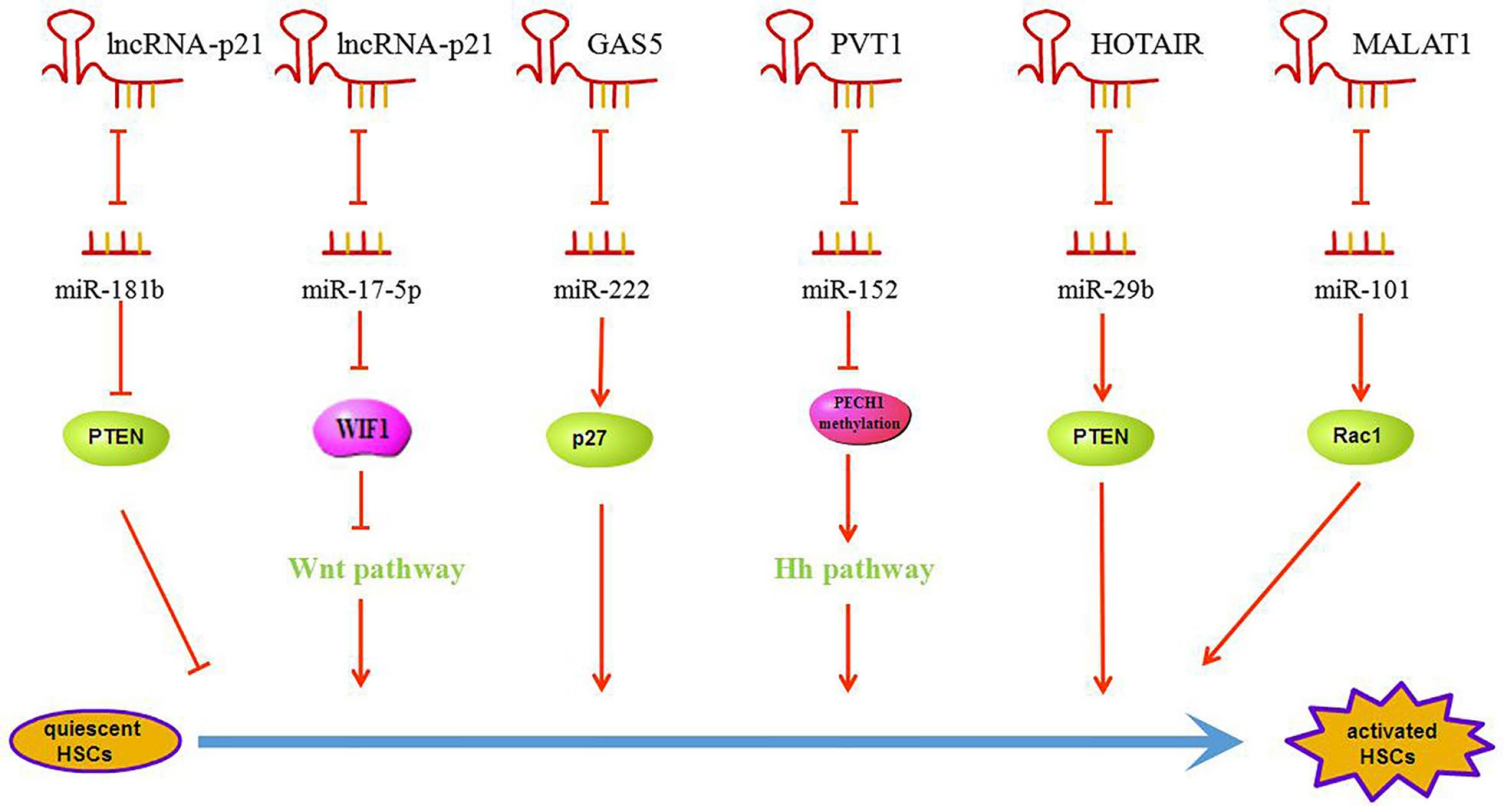
LncRNA represses miRNA to regulate the process of liver fibrosis. LncRNA-p21 inhibits the activation of HSCs through miR-181b and miR-17-5p; GAS5 inhibits the activation of HSCs through miR-222. PVT1, HOTAIR and MALAT1 promote the activation of HSCs through miR-152, miR-29b and miR-101 respectively

**Fig. 5:**
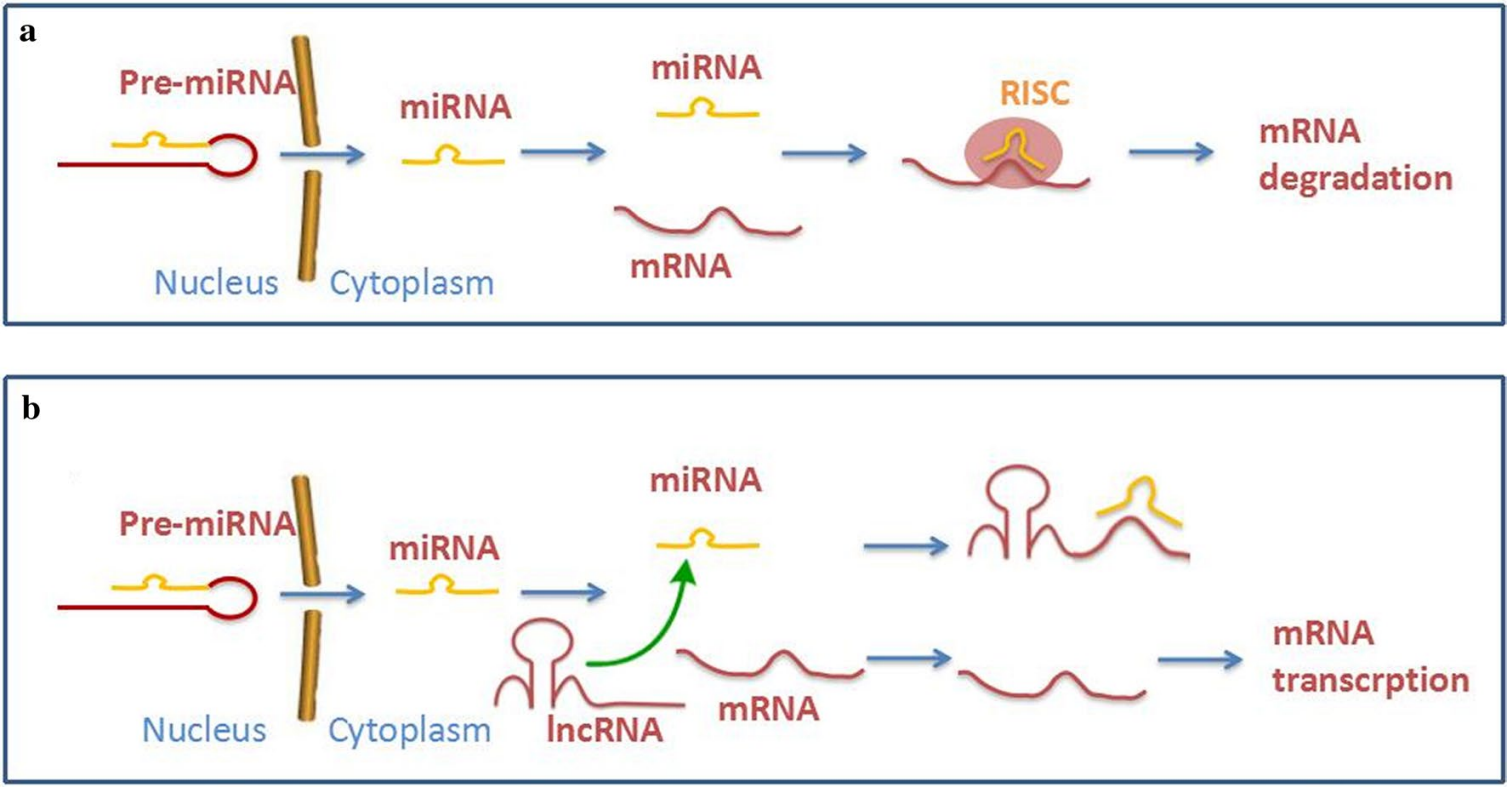
**a** The pre-miRNA gets out from the nucleus to be the mature miRNA. The mature miRNA incorporates into the RISC and the target mRNA to the target mRNA. **b** The activity of the miRNAs is inhibited by the presence of lncRNAs, which act as ceRNAs by sharing common MREs. Low levels of available miRNAs for the target mRNA translations

Whether ceRNA is an inhibitor or an activator to HSC activation depends on the spongy of miRNAs (Fig. 4). The model of how lncRNA works as ceRNA to sponge miRNAs may be widely accepted in (Fig. 5). The binding sites between lncRNA and miRNA shown in this review are displayed in the table (Table 1).

**Table 1:**
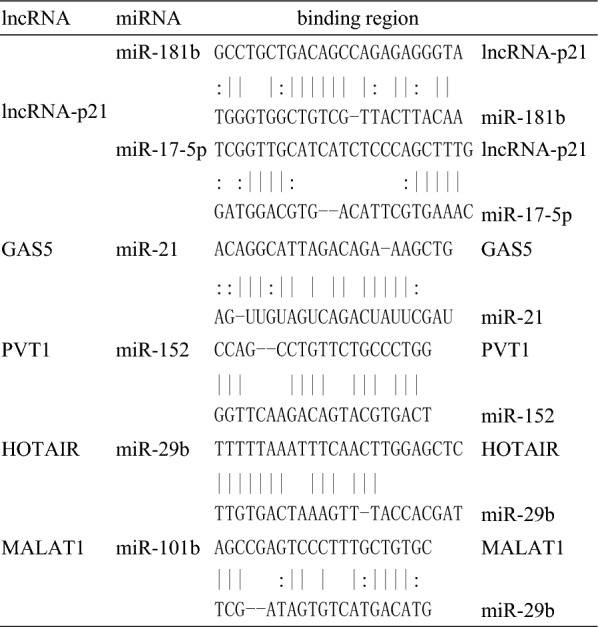
lncRNA as ceRNA in hepatic fibrosis

Only these lncRNAs that are described in this review are shown

## Conclusion

Attention has been paid to lncRNA structure, function and evolution. Although there is a great interest in new lncRNAs, it is a scientific topic in the future. To explore new therapies of hepatic fibrosis, more lncRNAs have been found to be involved in the activation of HSCs.

MALAT1 and lncRNA-LFAR1 activate HSCs through the TGF-β signaling pathway. Some lncRNAs will be discovered to induce the activation of HSCs via the TGF-β signaling pathway in the future. Other signaling pathways, such as Wnt, NF-κB and Notch signaling pathway, are also related with the HSC activation. The lncRNA AC067945.2 down-regulates collagen expression in skin fibroblasts and it possibly correlates with the VEGF and Wnt signalling pathways [81]. Therefore, it is worthwhile to explore some new lncRNAs via other signaling pathways that take part in the activation of HSCs.

MEG3 and HIF1A-AS1 inhabit the HSC activation with DNA methylation. MEG3 and HIF1A-AS1 are repressed by DNMT1 and TET3 respectively. MEG3 causes the accumulation and activation of p53 that decreases proliferation and increases apoptosis of activated HSCs. The mechanism of HIF1A-AS1 inhabiting the HSC activation is still need to be explored. It is also possible that DNMT1 or TET3 regulates other lncRNAs in the process of hepatic fibrosis. Thus, the interaction between lncRNAs and other enzymes associated with methylation is also worth studying in hepatic fibrosis.

The last role of lncRNAs regulating the HSC activation is to be ceRNAs for miRNAs. PVT1, HOTAIR and MALAT1 promote the activation of HSCs. LncRNA-p21 and GAS5 reduce the activation of HSCs. The miRNAs are inhibited by the lncRNAs which act as ceRNAs via sharing common MREs. The interaction between lncRNAs and miRNAs is not a one-to-one relationship, for example, lncRNA-p21 represses miR-181b and miR-17-5p. In addition, lncRNA MIR100HG has been confirmed to encode miR-100, let-7a-2 and miR-125b-1. It is worth exploring whether any lncRNAs regulate miR-cluster in liver fibrosis or not.

The differences in the expression of lncRNA between normal and hepatic fibrotic tissues not only imply that lncRNAs may take part in the progression of the hepatic fibrosis, but also suggest that lncRNAs may be the biomarkers for the clinical diagnosis of hepatic fibrosis. Moreover, it is possible that not only lncRNAs itself, but also both the binding proteins and the target genes will be new therapeutic targets, which may lead to the development of new anti-fibrosis treatments.

## Abbreviations

lncRNAs: long non-coding RNAs
ECM: extracellular matrix
HSCs: hepatic stellate cells
ceRNAs: competing endogenous RNAs
TGF-β: transforming growth factor-β
MALAT1: metastasis-associated lung adenocarcinoma transcript 1
SIRT1: silent information regulator 1
lnc-LFAR1: liver fibrosis-associated lncRNA1
SBE: Smad2/3 binding sites
DNMT: DNA methyltransferases
TET: ten–eleven translocation methylcytosine dioxygenase
MEG3: maternally expressed gene 3
α-SMA: α-smooth muscle actin colIα1: alpha-1 type I collagen
miRNAs: MicroRNAs
HIF1A-AS1: lncRNA HIF 1 alpha-antisense RNA 1
lncRNA-p21: long intergenic non-coding RNA-p21
PTEN: the phosphatase and tensin homologue deleted on chromosome 10
GAS5: growth arrest-specific transcript 5
PVT1: plasmacytoma variant translocation 1
HOTAIR: Homeobox (HOX) transcript antisense RNA
MREs: miRNA response elements
